# Inhibition of miR-128 Enhances Vocal Sequence Organization in Juvenile Songbirds

**DOI:** 10.3389/fnbeh.2022.833383

**Published:** 2022-02-25

**Authors:** Caitlin M. Aamodt, Stephanie A. White

**Affiliations:** ^1^Department of Integrative Biology and Physiology, University of California, Los Angeles, Los Angeles, CA, United States; ^2^Department of Pediatrics, University of California San Diego School of Medicine, La Jolla, CA, United States

**Keywords:** microRNA, songbird, neurodevelopment, learning, memory, stereotypy

## Abstract

The molecular mechanisms underlying learned vocal communication are not well characterized. This is a major barrier for developing treatments for conditions affecting social communication, such as autism spectrum disorder (ASD). Our group previously generated an activity-dependent gene expression network in the striatopallidal song control nucleus, Area X, in adult zebra finches to identify master regulators of learned vocal behavior. This dataset revealed that the two host genes for microRNA-128, ARPP21 and R3HDM1, are among the top genes whose expression correlates to how much birds sing. Here we examined whether miR-128 itself is behaviorally regulated in Area X and found that its levels decline with singing. We hypothesized that reducing miR-128 during the critical period for vocal plasticity would enhance vocal learning. To test this, we bilaterally injected an antisense miR-128 construct (AS miR-128) or a control scrambled sequence into Area X at post-hatch day 30 (30 d) using sibling-matched experimental and control pupils. The juveniles were then returned to their home cage and raised with their tutors. Strikingly, inhibition of miR-128 in young birds enhanced the organization of learned vocal sequences. Tutor and pupil stereotypy scores were positively correlated, though the correlation was stronger between tutors and control pupils compared to tutors and AS miR-128 pupils. This difference was driven by AS miR-128 pupils achieving higher stereotypy scores despite their tutors’ lower syntax scores. AS miR-128 birds with tutors on the higher end of the stereotypy spectrum were more likely to produce songs with faster tempos relative to sibling controls. Our results suggest that low levels of miR-128 facilitate vocal sequence stereotypy. By analogy, reducing miR-128 could enhance the capacity to learn to speak in patients with non-verbal ASD. To our knowledge, this study is the first to directly link miR-128 to learned vocal communication and provides support for miR-128 as a potential therapeutic target for ASD.

## Introduction

The zebra finch songbird is an important model for understanding the cellular and molecular basis of learned vocal communication. Songbirds and humans share cortico-striato-thalamic circuits with convergently specialized gene expression patterns that underlie learned vocal communication (Pfenning et al., 2014). Young male zebra finches learn to communicate a unique courtship song during a developmental critical period, and after this plasticity window closes it is much more difficult for a bird to modify his song (Aamodt et al., 2020). During this period, young males learn to sing sequences of individual syllables that are gradually organized into patterns called “motifs.” Females of this species do not sing but may respond to a courtship song with innate calls. Female zebra finches strongly prefer stereotyped motifs compared to song with more variable organization (Woolley and Doupe, 2008), and young birds put forth effort to hone their songs.

Since the sequencing of the zebra finch genome (Warren et al., 2010) it has become apparent that many genes that affect social communication in humans are also dynamically regulated when a bird sings (Hilliard et al., 2012; Burkett et al., 2018). Previous work from our group identified modules of genes that are co-regulated by singing (Hilliard et al., 2012). Within this dataset Activity-Regulated Phosphoprotein 21 (ARPP21) and R3H Domain Containing 1 (R3HDM1) are two of the top genes most strongly correlated to singing. ARPP21 and R3HMD1 are host genes for microRNA-128. This led us to hypothesize that miR-128 could be a key regulator of activity-dependent gene expression in learned vocalization.

microRNAs are small (21-22 nucleotide) sequences that typically regulate gene transcripts. One of the most highly expressed microRNAs in the brain, miR-128, is elevated in postmortem cerebellar tissue from autism spectrum disorder (ASD) patients and is among the top ten microRNAs in a co-regulated module abberantly upregulated in postmortem ASD cortex (Wu et al., 2016), suggesting that it plays a role in vocal communication. This microRNA regulates both neural proliferation and neuronal migration (Bruno et al., 2011; Franzoni et al., 2015). Loss of Fragile-X mental retardation protein (FMRP) leads to elevation of miR-128 (Men et al., 2020), indicating that increased miR-128 may be a convergent mechanism shared between idiopathic and syndromic forms of ASD. Prior studies linking miR-128 to cognition and behavior further support a role for miR-128 in human and songbird vocal learning. In the mouse infralimbic prefrontal cortex miR-128 inhibition decreased fear memory extinction, whereas miR-128 overexpression promoted the extinction of the memory (Lin et al., 2011). miR-128 levels in the striatum also regulate motor behavior (Tan et al., 2013). Lower miR-128 levels were associated with greater neuronal excitability and increased motor activity, whereas higher levels suppressed neuronal activity and associated motor behavior. The authors attributed these findings to miR-128-driven changes in ion channels as well as in the Extracellular Regulated Kinase 2 (ERK2) pathway, which is also important for birdsong (Burkett et al., 2018) and ASD (Gazestani et al., 2019).

Additional support for the role of miR-128 in social vocalization comes from rodent studies of ultrasonic vocalization (USV), a behavior commonly studied in rodent pups who have been isolated from their mother. Although to our knowledge USVs have not been studied in mice with miR-128 directly modified, there are several rodent studies where reduction of miR-128 target genes linked to ASD led to deficits in vocal communication. One such gene is Reelin (RELN), a secreted glycoprotein that plays an important role in neurodevelopment. RELN is a direct target of miR-128 [Evangelisti et al., 2009; Lin et al., 2011; Rev in Ching and Ahmad-Annuar (2015)]. In mice, reduction or loss of RELN leads to delays in both motor and vocal development (Ognibene et al., 2007; Romano et al., 2013). Loss of RELN’s target receptors very low density lipoprotein receptor (VLDLR) and disabled 1 (DAB1) also lead to deficits in neonate USVs (Fraley et al., 2016), suggesting that this pathway plays an important role in vocal communication.

Another interesting line of evidence comes from *in utero* exposure models of developmental disabilities. These include prenatal exposure to agents that trigger inflammation to mimic maternal infection, such as LPS [Rev in Premoli et al. (2021)], and pharmaceuticals such as valproate (Gzielo et al., 2020). Exposure to these agents *in utero* leads to reduced USVs in offspring. Although LPS-induced levels of miR-128 in rodents have not been directly measured, studies in multiple cell lines suggest that LPS dramatically increases miR-128 levels (Shyamasundar et al., 2018; Xie et al., 2021). Valproate has also been shown to increase miR-128 in cultured human neurons (Kidnapillai et al., 2020), suggesting that this molecular mechanism could be common to ASD emerging from diverse etiologies. However, the role of miR-128 in learned vocal communication has yet to be explored.

Here we use a small interfering RNA (siRNA) antisense construct to inhibit miR-128 in the striatopallidum of developing zebra finches. We show that inhibition of miR-128 leads to enhanced motif sequence stereotypy 45 days after surgery relative to controls that received a scramble sequence. We then characterize additional effects of miR-128 inhibition on spectral and temporal features of song. Our results indicate that miR-128 plays an important role in learned vocal communication and may thus be a viable therapeutic target for disorders of social communication where miR-128 is aberrantly elevated, including ASD.

## Materials and Methods

### Enrichment Map

1,215 predicted miR-128 targets from TargetScan (Agarwal et al., 2015) were analyzed for pathway enrichment using g:Profiler (Raudvere et al., 2019), including the biological processes of Gene Ontology (Ashburner et al., 2000; The Gene Ontology Consortium, 2021) and molecular pathways of Reactome (Jassal et al., 2020). The resulting Generic Enrichment Map file was visualized using the Enrichment Map application (Merico et al., 2010) in Cytoscape (Shannon et al., 2003).

### Hypergeometric Overlap Gene Enrichment Test

**FIGURE 1:**
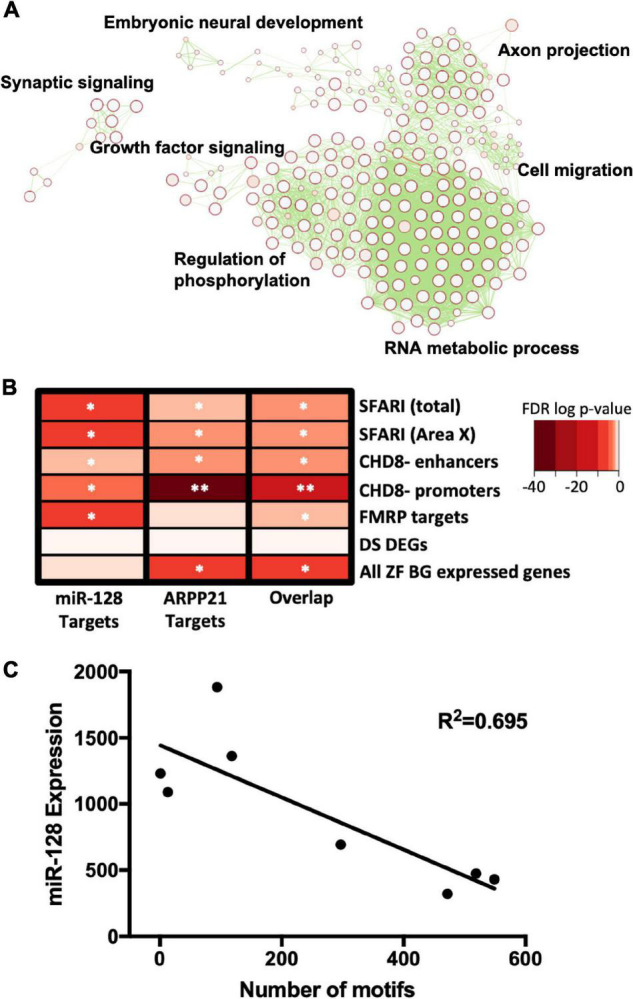
**(A)** Predicted miR-128 targets were analyzed for pathway enrichment and then clustered by similarity to generate an enrichment map. Cluster annotations are summarized with adjacent labels in black. **(B)** miR-128 targets are enriched in ASD-related gene datasets. Heat map shows gene enrichment analysis for miR-128 target genes, ARPP21 target genes, or both [Overlap] in autism-related gene lists: all autism risk genes from the Simons Foundation Autism Research Initiative (SFARI) database [SFARI (total)], SFARI genes expressed in Area X [SFARI (Area X)], CHD8 targets at enhancers [CHD8- enhancers] and promoters [CHD8- promoters], Fragile-X mental retardation protein target genes [FMRP targets]. Neural genes differentially expressed in Down syndrome [DS DEGs] and all genes expressed in the adult zebra finch basal ganglia [All ZF BG expressed genes] are included as negative controls. FDR corrected *p*-value indicated by *p < 5.00E–02, **p < 5.00E–12. **(C)** Activity-dependent regulation of miR-128 in adult zebra finch Area X as measured by qPCR. miR-128 undergoes activity-dependent regulation in the vocal communication region of the basal ganglia in adult songbirds (*R*^2^ = 0.695, *p* = 0.01). miR-128 levels were normalized to loading control U6 snoRNA.

Gene enrichment analysis (Figure 1A) was performed to identify lists of genes enriched in predicted miR-128 targets using the GeneOverlap package in R.^1^ The Simons Foundation Autism Research Initiative (SFARI Gene) database provided a comprehensive list of ASD candidate genes (Abrahams et al., 2013).^2^ The analysis included all available genes from the SFARI database and SFARI genes found in adult songbird Area X (Hilliard et al., 2012; Burkett et al., 2018). We also looked for overlap between genes affected by high effect size mutations that cause ASD and miR-128 targets. These included chromodomain-helicase-DNA-binding protein 8 targets at enhancers [CHD8- enhancers] and promoters [CHD8- promoters] in human fetal cortex (Cotney et al., 2015), and Fragile-X mental retardation protein target genes [FMRP] from P11–P25 mouse whole brain extracts (Darnell et al., 2011). We also included genes differentially expressed in Down Syndrome [Down Syndrome] postmortem brain using samples from multiple regions spanning mid-fetal development to adulthood (Olmos-Serrano et al., 2016) as a negative control. As a second control we included all genes expressed in adult songbird Area X. In these datasets we tested for enrichment in predicted miR-128 target genes, mRNA binding protein (and miR-128 host gene), ARPP21 target genes, and genes targeted by both miR-128 and ARPP21 (Rehfeld et al., 2018). The background was limited to 14,313 songbird brain-expressed genes. Statistically significant gene overlap was calculated using Fisher’s Exact Test followed by FDR correction.

### microRNA Assay

Upon extraction whole brains were immediately flash frozen using liquid nitrogen and stored at −80°C until processing. Brains were then allowed to raise to −20°C in the cryostat, then mounted whole to a chuck using TissueTek (Sakura) mounting medium. Brains were sectioned coronally until reaching the beginning of Area X. Tissue punches were collected from Area X using a 20 gauge Luer adapter inserted 1 mm deep. Total RNA (including small RNA) was isolated using a TRIzol Reagent kit (Zymo Research). Taqman MicroRNA Assays specific for miR-128 (assay ID: 002216) and U6 snRNA (assay ID: 001973) as a loading control were used to reverse- transcribe microRNA using and the TaqMan microRNA reverse transcription kit (Applied Biosystems, Waltham, MA, United states). Five ng of RNA were added to each 15 uL reverse transcription reaction. Expression was analyzed using the TaqMan microRNA assay kit (Applied Biosystems, Waltham, MA, United states) with TaqMan Fast Advanced Master Mix according to the manufacturer’s instructions. 1.33 uL cDNA template, or nuclease free water for no template controls, was added to each well. All reactions were run in triplicate. The thermal protocol was set to one cycle at 50°C for 2 min for UNG activation, one cycle at 95°C for 20 s for enzyme activation, then 40 cycles at 95°C for 1 s to denature followed by 20 s at 60°C for annealing/extension. miR-128 levels were normalized to U6 as a loading control. Liver small RNAs were used as a negative control, since miR-128 is expressed at very low levels (Luo et al., 2012).

### Subjects

Subjects were male zebra finches (*Taeniopygia guttata*). To measure activity-dependent regulation of gene expression adult birds (>125 d) were allowed to sing in a sound attenuation chamber for 2 h prior to tissue collection by cervical dislocation and brain extraction. A total of eight birds were used. For the miR-128 inhibition experiments juvenile birds beginning at 30 days post-hatch (30 d) were selected for surgery and subsequently raised to 75 d. A total of 20 birds underwent stereotaxic neurosurgeries targeting Area X bilaterally (see below). Ten birds from seven breeding pairs were treated by focal injection of an AAV bearing a miR-128 sponge sequence and 10 siblings were treated with a scrambled sequence as a control. Birds were primarily housed in home cages with parents and siblings, unless being recorded individually in a sound attenuation chamber. The vivarium and recording chambers are humidity- and temperature-controlled (22°C) and on a 12 h light: dark cycle with half hour “dusks” and “dawns.” Birdseed, water, millet, cuttlebone, and grit were provided *ad libitum*. Baths, hardboiled egg, and vegetables were provided weekly. Animal use was in accordance with the Institutional Animal Care and Use Committee at the University of California, Los Angeles and complied with the American Veterinary Medical Association Guidelines.

### Experimental Timeline

**FIGURE 2:**
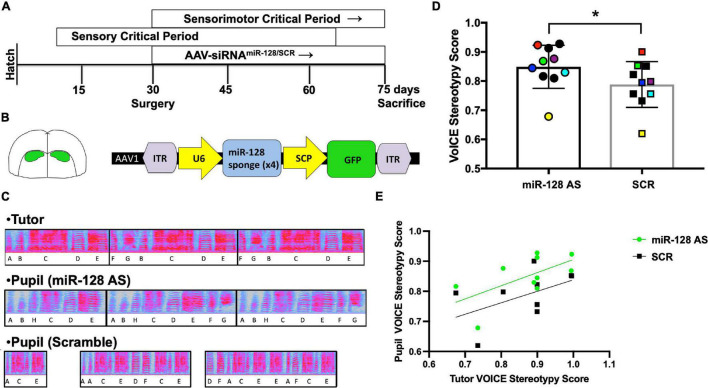
**(A)** Timeline of experimental procedures. Developmental critical periods are shown in top two boxes. **(B)** Region of interest (Area X) and miR-128 antisense viral construct. (Left) Diagram showing viral targeting of Area X. (Right) Schematic characterizing the antisense construct used to inhibit miR-128. Adeno-associated virus 1 vector (AAV1) with inverted terminal repeats to maintain construct integrity (ITR). The type III RNA polymerase III promoter U6 was used to drive expression of the miR-128 siRNA sponge. A proprietary supercore promotor designed by Rachael Neve was used to drive GFP. **(C)** Exemplar motifs from an adult male (Tutor), a juvenile son who received the miR-128 antisense construct [Pupil (miR-128 AS)], and another juvenile son from the same clutch who received the scramble control construct [Pupil (Scramble)]. **(D)** Analysis of song sequence organization in 75 d sibling-matched miR-128 AS and scramble control birds (*N* = 10/group), *p = 0.014, Wilcoxon signed-rank test. Symbol colors denote pupils that shared the same tutor. **(E)** Tutor and control pupil syntax stereotypy scores are positively correlated (*R*^2^ = 0.49, *p = 0.025), whereas the correlation between tutors and AS miR-128 pupils does not reach significance (*R*^2^ = 0.37, *p* = 0.061).

Experiments were performed as schematized in Figure 2A. Subjects were housed in their home cage with both parents except during short periods to record song individually. At 30 d surgeries were performed and the birds were allowed to recover in their home. At ∼70 d birds were returned to recording chambers for behavioral experiments. Birds were given a day or two to habituate to the chamber, recorded for undirected singing the next day, prevented from singing for 2 h and then allowed to sing and recorded for 2 h, allowed to sing normally for 1 day, and then the final recording was collected and the bird was sacrificed 2 h after lights on.

### siRNA Constructs and Viral Vectors

An siRNA sponge previously shown to inhibit miR128 function (Lin et al., 2011) and a scrambled control construct were adapted for use in zebra finches and cloned into an AAV1 viral vector (Virovek, Hayward, CA, United states). The sponge consisted of three “bulged” miR-128 binding site sequences separated by 4nt spacers. The scramble construct differed only in that the control sequence contained a standard scramble sponge provided by Virovek. Both sponge and scramble sequences were driven by the U6 promoter, as previously described (Haesler et al., 2007). Both viral titers were 10^13 vg/ml. 303.6 nl of virus were delivered to each hemisphere in six injections of 50.6 nl spaced 5 um apart.

### Stereotaxic Surgery

At 30 d, neurosurgeries were performed as described (Heston and White, 2015; Burkett et al., 2018). Briefly, subjects were administered preoperative meloxicam and then anesthetized using 2–4.5% isoflurane in oxygen. A custom-built avian stereotaxic apparatus was used to target Area X at the following coordinates: 45° head angle, 5.15 mm rostral of the bifurcation of the midsagittal sinus, 1.60 mm lateral of the midline, at a depth of 3.25 mm relative to the surface of the exposed brain. Virus was injected bilaterally using a Drummond Nanoject II *via* a glass micropipet (∼40 um inner diameter). Six 50.6 nL injections were performed in Area X at a depth of 3.25, 3.20, 3.15, 3.10, 3.05, and 3.0 mm to maximize the area transfected. Injections were performed with a 15 s pause to allow virus to disperse, with a final 5-min wait before removing the micropipet. The scalp incision was closed using Vetbond (3M, St. Paul, MN, United States), then covered with dental cement. Birds remained on oxygen for ∼2 min until alert, then returned to their home cages.

### Audio Recording

Recordings were collected in sound attenuation chambers. Songs were digitally recorded (Sound Analysis Pro; SAP; Tchernichovski et al., 2000) using a PreSonus FirePod or Audiobox (44.1 kHz sampling rate; 24-bit depth) and stored uncompressed.

### Sequence Stereotypy

Sequence stereotypy for juveniles and tutors was measured by calculating syllable transition probability using Vocal Inventory Clustering Engine [VoICE; (Burkett et al., 2015)].^3^ Syllables from the first 20 motifs were hand segmented and a SAP Feature Batch was generated, producing a table of the quantified values for all spectral features for each syllable (Tchernichovski et al., 2000). Motifs were defined as sequences of recurrently produced syllables comprised of at least three syllables, though all birds in this study produced greater than three syllables per motif. Syllables are defined as discrete acoustic elements (Yu and Margoliash, 1996). We segmented syllables at the milliseconds of silence that separate each syllable. In cases where two syllables were conjoined, we chose to lump into a single syllable or split into two syllables based on how they most frequently appeared in the bird’s song.”

Then VoICE was used to cluster syllables by similarity. Once syllable types were defined, VoICE was used to calculate syllable transition probability across all motifs in the order they were produced and generate a stereotypy score.

### NS-UD Paradigm

**FIGURE 3:**
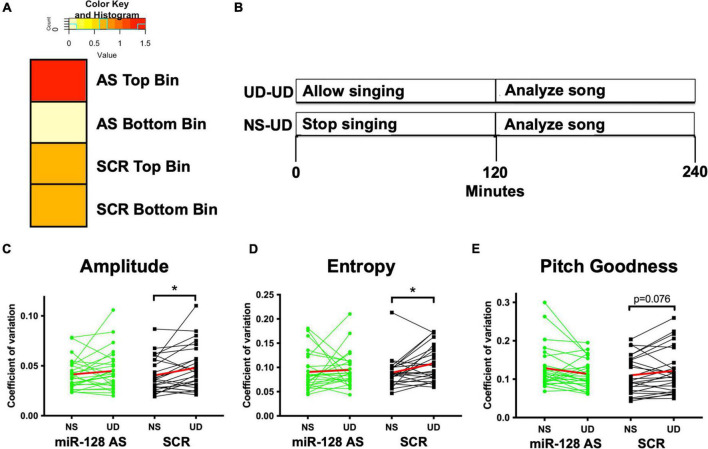
**(A)** Tempo data divided into two bins based on the syntax score each bird’s tutor. AS miR-128 birds from tutors with higher syntax scores had faster tempos (tempo score < 0.13, Odds Ratio 1.5) relative to AS miR-128 birds from tutors with lower syntax scores (OR 0), or SCR birds from both bins (OR 0.67). **(B)** Schematic illustrating the non-singing vs. singing (NS-UD) paradigm. On separate days a test subject is either allowed to sing by himself for 4 h (UD-UD) or is prevented from singing for 2 h and then allowed to sing for the subsequent 2 h (NS-UD). Our prior work revealed that song variability is positively correlated to the amount of time singing (Heston and White, 2015; Burkett et al., 2018). **(C–E)** Coefficient of variation (CV) for spectral features [**(C)** Amplitude, **(D)** Entropy, **(E)** Pitch Goodness] of song following 2 h of singing (UD) or non-singing (NS). The experimental AS miR-128 group is shown in green, and the control SCR group is in black. The average trendline is shown in red. Syllables from five AS miR-128 birds (*N* = 26) and five sibling-matched control SCR birds(*N* = 25) were analyzed, *p < 0.05 bootstrap *t*-test.

“NS-UD” experiments were performed as follows and as described in Figure 3B, as well as prior publications (Miller et al., 2010; Chen et al., 2013; Heston and White, 2015; Burkett et al., 2018). Briefly, on one morning, individual birds were distracted if they appeared to initiate singing during the first 2 h after lights on, then recorded to capture song after the non-singing (NS) state. No birds sang >10 motifs in this condition. Prior work shows that the gene expression profiles of NS birds are similar to those of birds that voluntarily did not sing in enclosed chambers, suggesting that this paradigm does not induce a stress response (Miller et al., 2010). NS songs were compared to songs from the same birds that were recorded on a different day following 2 h of singing in a single-housed chamber (undirected singing: UD). Syllables from the first 20 motifs sung in these two conditions were hand segmented and acoustic features were quantified in a SAP Feature Batch. Syllables were clustered using VoICE. To calculate the effect size, we used the formula (NS-UD)/NS + UD).

### Song Tempo

Tempo was defined as the number of syllables per motif divided by the duration of the motif in seconds. Tempo scores were averaged across the first 20 motifs for each bird. Tempo data was divided into two bins based on the syntax score each bird’s tutor. Since there was an odd number of tutors, the tutor with the median score had two pupils in each of the four bins based on their own scores. The final N included five birds in each bin, 20 birds total.

## Results

We began our investigation by broadly determining what functional roles miR-128 targets share. Since miR-128 and many of its targets are highly conserved in vertebrates (Bruno et al., 2011), we wanted to better understand what processes miR-128 regulates. Using predicted targets (Rehfeld et al., 2018) we generated an enrichment map to identify clusters of genes with shared functional roles (Figure 1A and Supplementary Table 1). Several key processes were implicated, including embryonic neural development, axon projection, cell migration, and growth factor signaling. Regulation of phosphorylation was also strongly implicated, suggesting converging effects on signaling enzyme cascades central to birdsong (Burkett et al., 2018) and autism (Gazestani et al., 2019).

We next investigated whether miR-128 targets would be enriched in gene lists relevant for human ASD using a hypergeometric gene overlap test (Figure 1B and Supplementary Table 2). miR-128 targets were enriched in the Simons Foundation Autism Research Initiative’s (SFARI) list of high confidence syndromic ASD genes (with a confidence score of 3 or greater), using both the entire list or restricting the list to those genes expressed in Area X. We also found that miR-128 targets were enriched in genes regulated by syndromic ASD gene chromodomain helicase DNA binding protein 8 (CHD8) at either its enhancers or promoters. Similarly, we found an enrichment of miR-128 targets among genes regulated by syndromic ASD gene and RNA binding protein Fragile X Mental Retardation Protein (FMRP). As a negative control, we checked for enrichment of miR-128 targets in Down Syndrome differentially expressed genes and found that they were not enriched, as expected. As an additional negative control, we checked for enrichment of miR-128 targets across all genes expressed in the adult zebra finch striatopallidum and did not find an enrichment. We interpret these results to suggest that autism severity is mediated by multiple hits that converge on shared pathways, and that one of these hits that influences other changes that predispose ASD is the level of miR-128 expression.

Next, we checked to see how the amount of singing, measured by total number of motifs produced during the first 2 h of lights-on, affects miR-128 expression in Area X (Figure 1C). Using tissue from eight adult (>120 d) males, we found that total number of motifs was negatively correlated to miR-128 levels (*R*^2^ = 0.695, *p* = 0.01). This suggests that miR-128 is downregulated in Area X in an activity-dependent manner.

To better understand how miR-128 influences learned vocal communication we injected an antisense miR-128 (AS miR-128) siRNA construct or a scramble control sequence into Area X (Figure 2A, *N* = 10 per group) of sibling matched pairs at 30 d, then raised the birds with both parents in their home cage. At 75 d we assessed how song quality was influenced (Figure 2B). Sequence stereotypy was measured by calculating syllable transition probability using Vocal Inventory Clustering Engine [VoICE; (Burkett et al., 2015); See text footnote 3]. Syllables were hand segmented and a SAP Feature Batch was generated, producing a table of the quantified values for all spectral features for each syllable (Tchernichovski et al., 2000). Then VoICE was used to cluster syllables by similarity. Once syllable types were defined, VoICE was used to calculate syllable transition probability and generate a stereotypy score. Strikingly, syllable sequence organization was significantly improved in juvenile birds who received the miR-128 antisense construct relative to sibling-matched controls raised under normal conditions (Figures 2C,D).

AS pupil sequence stereotypy scores correlated with their tutors’ scores (Figure 2E), although this correlation did not reach significance in AS miR-128 pupils due to higher syntax scores at the lower end of the score range. To better understand the ceiling effect for pupils at the higher end of the spectrum we calculated tempo for each pupil as previously described (Mets and Brainard, 2018). Overall, we found no differences in tempo between groups using a *t*-test with resampling or Wilcoxen rank sum test. However, when the birds were binned by tutor syntax score, birds in the higher bin for AS miR-128 were more likely to have faster tempos (OR = 1.5, Figure 3A). We also counted the total number of motifs produced over the course of 2 h in the 75 d miR-128 AS and SCR sibling pairs (Supplementary Figure 1). Although not significant, there was an interesting trend of miR-128 AS birds with siblings who sang a low number of motifs singing more than their counterparts, except for the birds whose SCR siblings sang the highest number of motifs. This suggests that there may be a limit to how much reducing miR-128 levels promotes song production. Future research should focus on birds with impoverished song to better characterize the potential therapeutic value of miR-128 inhibition.

To further characterize the effects of the miR-128 sponge on learned vocal behavior we employed the “NS-UD paradigm” as follows (Figure 3B; Miller et al., 2010; Chen et al., 2013; Heston and White, 2015; Burkett et al., 2018). When a bird sings by himself for 2 h in the morning (undirected) the spectral features of birdsong become more variable in correlation to the amount of time the bird has been singing (Miller et al., 2010). On 1 day a bird was recorded after 2 h of singing, while on a separate day the same bird was recorded after 2 h of non-singing. Analysis of the coefficient of variation for the spectral features on both days showed that birds who received the scrambled control had more variable song in the UD vs. NS condition, in keeping with previous findings. However, the miR-128 antisense birds did not show this same increase in spectral feature variability. Results reached significance for amplitude and entropy and approached significance for pitch goodness using a *t*-test with resampling (Figures 3C–E and Supplementary Figure 2).

Motif level analysis of syllable sequence stereotypy did not uncover differences between the NS and UD conditions; however, it did replicate the results in Figure 2D with independent data sets, showing that the result was consistent across days and robust even after 2 h of singing when song is typically less stereotyped (Supplementary Figure 3).

## Discussion

Here we show that inhibition of miR-128 during juvenile vocal development enhances song stereotypy. We did this by injecting a miR-128 antisense construct delivered *via* an AAV1 vector in Area X of 30 d birds and analyzing song at 75 d relative to sibling matched controls who received a construct with a scrambled sequence. Overall stereotypy scores for all pupils were correlated to their tutors’ scores, as expected. Strikingly, inhibition of miR-128 consistently led to enhancements in sequence stereotypy relative to sibling-matched controls. AS miR-128 birds from tutors with high stereotypy scores were more likely to produce songs with faster tempos. Although SCR controls showed more variability in spectral features after 2 h of singing in solitude, as is typical in this species, AS miR-128 birds did not show a typical loss of stereotypy in this time frame.

Zebra finches are the key model system for studying how activity-dependent gene regulation underlies developmental vocal communication learning (e.g., Haesler et al., 2007; Heston and White, 2015). Mechanisms that limit or constrain plasticity for vocal learning are of particular interest, because premature closure could be a major contributor to communication deficits in humans. Consistent with previous results, we found that miR-128 was highly expressed in zebra finch brain (Figure 1C; Luo et al., 2012), similarly to humans (Bruno et al., 2011). We show that reducing miR-128, which is aberrantly elevated in ASD patients, improves complex vocal sequences in 75 d zebra finches (Figure 2). These results show that miR-128 plays a role in constraining learned vocal communication, and thus miR-128 may be a promising pharmacotherapeutic target for treating ASD.

Our work is in keeping with previous studies showing that microRNAs regulate transcription in learned vocal communication. Shi and colleagues found that miR-9 and miR-140-5p are upregulated when a bird practices his song by himself and regulate key targets important for learned vocalization, including the speech and language-related ForkheadboxP2 transcription factor (FoxP2; Shi et al., 2013; Shi et al., 2018). Similarly, Larson et al. (2015) characterized changes in microRNAs associated with seasonal song plasticity in Gambel’s white-crowned sparrows. They found that miR-212 and miR-132 were key regulators of seasonal plasticity, which is interesting given that these microRNAs, like miR-128, are also aberrantly elevated in ASD (Wu et al., 2016). Within the auditory forebrain, microRNAs are regulated by passive listening to conspecific song (Gunaratne et al., 2011). Several studies have also identified sex differences in songbird microRNA regulation (Luo et al., 2012; Lin et al., 2014).

In rodents and human cells, miR-128 has been linked to both neuroplasticity and neurodevelopment. That leads us to think that here miR-128 may be regulating ongoing postnatal neurogenesis in Area X. Interestingly, songbirds and humans share ongoing basal ganglia neurogenesis in adulthood (Vellema et al., 2010; Thompson et al., 2013; Ernst et al., 2014), so this adaptation may be common to vocal learning species.

Interestingly, in the birds that received the AS miR-128 construct we did not see changes in the NS-UD paradigm that are typically associated with prolonged singing (Figure 3). This raises interesting questions about the evolution of learned vocal communication. Perhaps the evolution of stereotyped vocal sequence production starts with a smaller window when an organism can perform this behavior, and then evolution selects for individuals with greater endurance for producing learned vocalizations in a precise manner. Although some variability is necessary for any form of motor learning (Fee, 2014), it may be that the required changes can be more subtle and decrease as the bird learns, similarly to how juvenile birds produce more variable song than adults. It was recently discovered that miR-128 has undergone positive selection in the human lineage (Wang et al., 2020). In humans, multiple single nucleotide polymorphisms that reduce the expression of ARPP21 correlate with higher intelligence scores (Savage et al., 2018). Altogether, this suggests that miR-128 may be a key for understanding the evolution of language.

## Conclusion

In conclusion, these results reveal that expression levels of miR-128 in the basal ganglia directly affect sequence stereotypy in learned vocal communication. This study provides evidence that therapeutically targeting miR-128 during juvenile development has beneficial effects on vocal learning in an animal model of speech and language. Inhibition of miR-128 may be a safe approach for future therapeutic design for children with ASD and other developmental disabilities that impair speech and language development.

## Data Availability Statement

The raw data supporting the conclusions of this article will be made available by the authors, without undue reservation.

## Ethics Statement

The animal study was reviewed and approved by the Institutional Animal Care and Use Committee at the University of California, Los Angeles and complied with the American Veterinary Medical Association Guidelines.

## Author Contributions

CA and SW conceived and designed the study. CA coordinated and carried out all behavioral components and the molecular work, performed the statistical analysis, and drafted the manuscript. SW helped draft the manuscript. Both authors gave final approval for publication.

## Conflict of Interest

The authors declare that the research was conducted in the absence of any commercial or financial relationships that could be construed as a potential conflict of interest.

## Publisher’s Note

All claims expressed in this article are solely those of the authors and do not necessarily represent those of their affiliated organizations, or those of the publisher, the editors and the reviewers. Any product that may be evaluated in this article, or claim that may be made by its manufacturer, is not guaranteed or endorsed by the publisher.

## Footnotes

1
https://github.com/shenlab-sinai/geneoverlap

2
gene.sfari.org

3
https://github.com/zburkett/VoICE

## Supplementary Material

Supplementary Figure 1 **(A–H)** Coefficient of variation (CV) for spectral features [**(A)** Duration, **(B)** Amplitude, **(C)** Pitch, **(D)** Frequency Modulation, **(E)** Amplitude Modulation, **(F)** Entropy, **(G)** Pitch Goodness, **(H)** Mean Frequency] of song following 2 h of singing (UD) or non-singing (NS). The experimental AS miR-128 group is shown in green, and the control SCR group is in black. The average trendline is shown in red. Syllables from five AS miR-128 birds (*N* = 26) and five sibling-matched control SCR birds (*N* = 25) were analyzed.

Supplementary Figure 2 Motif level analysis of syllable sequence stereotypy in NS and UD conditions.

Supplementary Figure 3 Number of motifs produced during 2 h of singing in 75 d miR-128 AS and SCR controls. Symbol colors denote pupils that shared the same tutor.

Supplementary Table 1 Gene set enrichment analysis results used to generate Figure 1A.

Supplementary Table 2 Gene lists used to generate Figure 1B.
