# Supplementary figures and images for: Inhibition of miR-128 Enhances Vocal Sequence Organization in Juvenile Songbirds
Source: Front Behav Neurosci. 2022 Feb 25;16:833383. doi: 10.3389/fnbeh.2022.833383 (PMC8914539; doi:10.3389/fnbeh.2022.833383)

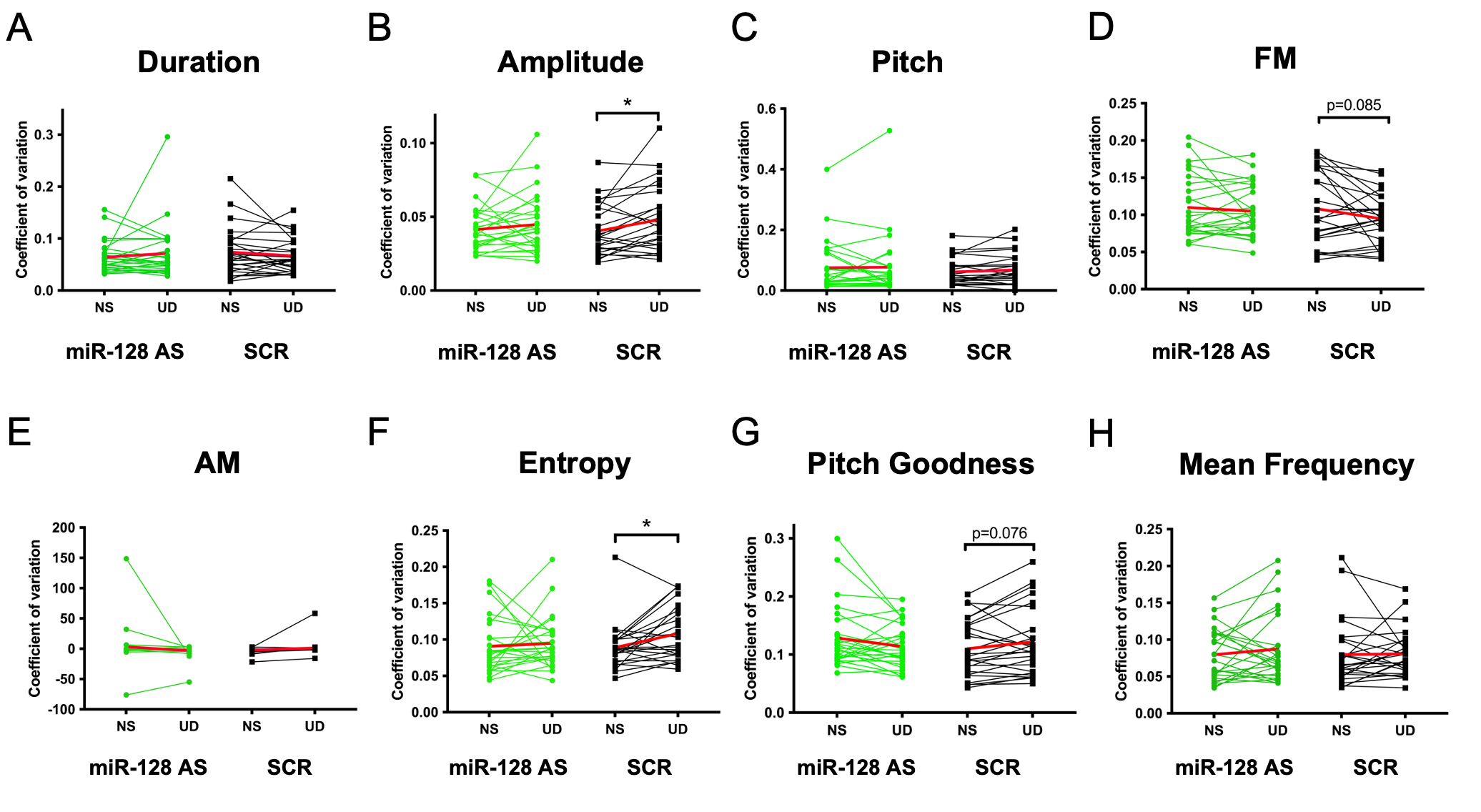

Supplement: Supplementary Figure 1 — (A–H) Coefficient of variation (CV) for spectral features [(A) Duration, (B) Amplitude, (C) Pitch, (D) Frequency Modulation, (E) Amplitude Modulation, (F) Entropy, (G) Pitch Goodness, (H) Mean Frequency] of song following 2 h of singing (UD) or non-singing (NS). The experimental AS miR-128 group is shown in green, and the control SCR group is in black. The average trendline is shown in red. Syllables from five AS miR-128 birds (N = 26) and five sibling-matched control SCR birds (N = 25) were analyzed. [file Image_1.tiff]

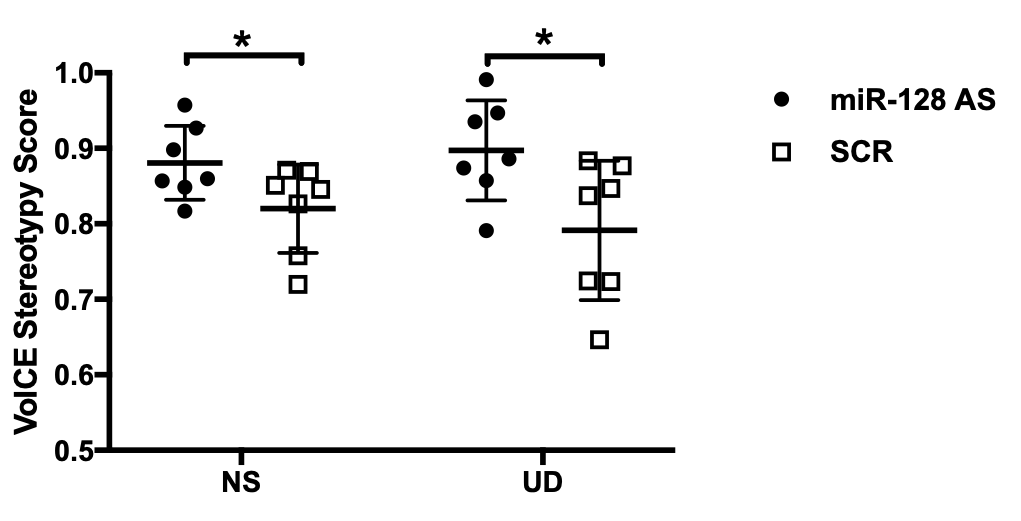

Supplement: Supplementary Figure 2 — Motif level analysis of syllable sequence stereotypy in NS and UD conditions. [file Image_2.tiff]

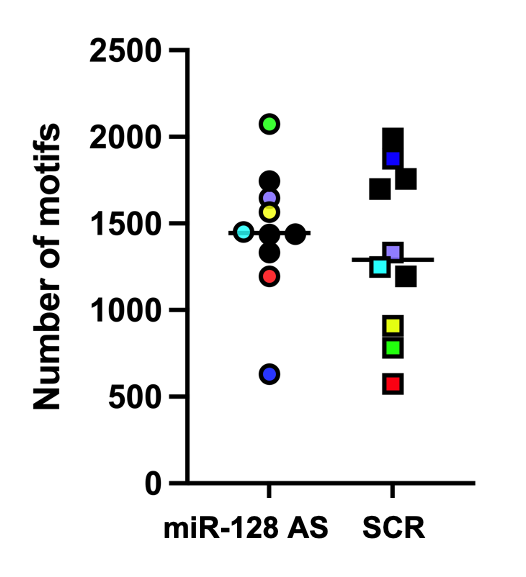

Supplement: Supplementary Figure 3 — Number of motifs produced during 2 h of singing in 75 d miR-128 AS and SCR controls. Symbol colors denote pupils that shared the same tutor. [file Image_3.tiff]
